# Cardiac Inflammation, Oxidative Stress, Nrf2 Expression, and Coagulation Events in Mice with Experimental Chronic Kidney Disease

**DOI:** 10.1155/2021/8845607

**Published:** 2021-01-16

**Authors:** Abderrahim Nemmar, Suhail Al-Salam, Sumaya Beegam, Nur Elena Zaaba, Javed Yasin, Naserddine Hamadi, Badreldin H. Ali

**Affiliations:** ^1^Department of Physiology, College of Medicine and Health Sciences, United Arab Emirates University, P.O. Box 17666, Al Ain, UAE; ^2^Zayed Center for Health Sciences, United Arab Emirates University, Al Ain, UAE; ^3^Department of Pathology, College of Medicine and Health Sciences, United Arab Emirates University, P.O. Box 17666, Al Ain, UAE; ^4^Department of Internal Medicine, College of Medicine and Health Sciences, United Arab Emirates University, P.O. Box 17666, Al Ain, UAE; ^5^Department of Life and Environmental Sciences, College of Natural and Health Sciences, Zayed University, P.O. Box 144534, Abu Dhabi, UAE; ^6^Department of Pharmacology and Clinical Pharmacy, College of Medicine & Health Sciences, Sultan Qaboos University, P.O. Box 35, Muscat, 123 Al-Khod, Oman

## Abstract

Chronic kidney disease (CKD) is known to be associated with cardiovascular dysfunction. Dietary adenine intake in mice is also known to induce CKD. However, in this experimental model, the mechanisms underlying the cardiotoxicity and coagulation disturbances are not fully understood. Here, we evaluated cardiac inflammation, oxidative stress, DNA damage, and coagulation events in mice with adenine (0.2% *w*/*w* in feed for 4 weeks)-induced CKD. Control mice were fed with normal chow for the same duration. Adenine increased water intake, urine output, relative kidney weight, the plasma concentrations of urea and creatinine, and the urinary concentrations of kidney injury molecule-1 and neutrophil gelatinase-associated lipocalin. It also decreased the body weight and creatinine clearance, and caused kidney DNA damage. Renal histological analysis showed tubular dilation and damage and neutrophilic influx. Adenine induced a significant increase in systolic blood pressure and the concentrations of troponin I, tumor necrosis factor-*α*, and interleukin-1*β* in heart homogenates. It also augmented the levels of markers of lipid peroxidation measured by malondialdehyde production and 8-isoprostane, as well as the antioxidants superoxide dismutase and catalase. Immunohistochemical analysis of the hearts showed that adenine increased the expression of nuclear factor erythroid-derived 2-like 2 by cardiomyocytes. It also caused cardiac DNA damage. Moreover, compared with the control group, adenine induced a significant increase in the number of circulating platelet and shortened the thrombotic occlusion time in pial arterioles and venules *in vivo*, and induced a significant reduction in the prothrombin time and activated partial thromboplastin time. In conclusion, the administration of adenine in mice induced CKD-associated cardiac inflammation, oxidative stress, Nrf2 expression, and DNA damage. It also induced prothrombotic events *in vivo*. Therefore, this model can be satisfactorily used to study the cardiac pathophysiological events in subjects with CKD and the effect of drug treatment thereon.

## 1. Introduction

There is a worldwide substantial increase in the prevalence of chronic kidney disease (CKD) attaining as much as 13%, and more than seven million people with end-stage kidney disease are requiring renal replacement therapy [1, 2]. The latter has been associated with the increase of prevalence of ageing, obesity, diabetes mellitus, hypertension, and metabolic syndrome [1, 2].

Kidney and cardiovascular diseases are tightly interconnected, and injury in one of these organs leads to adverse effects on the other one [1, 2]. In fact, it is well established that patients with CKD present cardiovascular complications including hypertension, thromboembolic disorders, cardiac hypertrophy and failure, and their pervasiveness augments with deteriorating kidney function [1, 2].

Animal models of CKD have been shown by several workers to be extrapolatable and useful (albeit imperfect) to the human disease [3]. In order to provide biological plausibility and better understanding on the mechanisms underlying CKD and their extrarenal impact, animal models with CKD are often utilized including the adenine-induced CKD [4]. When adenine is ingested by rodents, it is oxidized to 2,8-dihydroxyadenine by xanthine oxidase, which produces precipitates and crystals in the renal tubules which consequently induce tubular damage, inflammation, and fibrosis [5, 6].

Rats treated with adenine-induced CKD have been shown to develop cardiovascular complications including increase of systolic blood pressure (SBP) starting at week four, and at week 16, the SBP is further augmented and is associated with left ventricular hypertrophy and interstitial and perivascular inflammation and fibrosis and compromised vascular reactivity [4, 7, 8]. However, the mechanisms of action of cardiovascular injury in adenine-induced CKD are not fully understood.

Moreover, a recent study has shown a prolongation in tail bleeding time and delay in thrombus formation in cremaster arterioles following vascular injury in mice with CKD induced by 5/6^th^ nephrectomy- or adenine (0.25% for two weeks)-induced CKD [9]. On the contrary, other studies have reported platelet hyperactivity and increased thrombogenicity in a rat or mouse model of CKD induced by 5/6 ablation/infarction [10, 11].

We have recently shown that administration of adenine (0.2% *w*/*w* in feed for 4 weeks) in mouse-induced CKD is accompanied by lung oxidative stress, DNA damage, and fibrosis [12]. However, the impact of the latter model of CKD on the cardiovascular system has received only scant attention [4]. Therefore, the aim of this study conducted in mice was twofold: (1) to assess the effects of adenine (0.2% *w*/*w* in feed for 4 weeks)-induced CKD on SBP, heart histology, inflammation, oxidative stress, nuclear factor erythroid 2-related factor 2 (Nrf2) expression, and DNA damage and (2) to evaluate the impact of the adenine-induced CKD on circulating platelets, photochemically induced thrombosis in pial microvessels *in vivo* and prothrombin time (PT), and activated partial thromboplastin time (aPTT) *in vitro*.

## 2. Material and Methods

### 2.1. Experimental Animals and Treatments

Male C57BL/6 mice aged between 8 and 10 weeks, weighing in the beginning about 20-25 g (UAEU, College of Medicine and Health Sciences animal house), were housed at the Animal House of the College of Medicine and Health Sciences, UAEU, in light (12 h light : 12 h dark cycle), relative humidity of 50–60%, and temperature-monitored (21 ± 2°C) rooms. Animals had unrestricted access to tap water and commercial laboratory chow. They were indiscriminately separated into two groups of mice and put in cages. The control mice were given standard food for four weeks. The second group consisting of the adenine-treated group received the same diet in the form of powder containing adenine 0.2% *w*/*w* (0.2 g of adenine in 100 g of powder diet) for four weeks. The dose and duration of adenine treatment used in the present study were selected from our previous publications and have been shown to be effective in causing CKD in mice [6, 12–15]. It has been shown that when adenine is consumed by mice or rats, it gets metabolized to 2,8-dihydroxyadenine, which precipitates and produces tubular crystals that consequently induce kidney injury [5, 6, 16]. The weights of the animals were taken at the start of the study and just prior to sacrifice. Mice were relocated in metabolic cages on day 28 and kept there for 24 h to allow the quantification of water intake and urine volume. Twenty-four hours later, numerous renal and cardiovascular parameters were assessed. Mice were cared for under the protocol of the Animal Research Ethics Committee of our college and as per the NIH *Guide for the Care and Use of Laboratory Animals*, NIH publication no. 85-23, 1985.

### 2.2. Measurement of SBP

A computerized noninvasive tail-cuff manometry system was used to assess SBP in control and adenine-treated mice (ADInstruments, Colorado Springs, USA) [17]. To circumvent technique-induced stress, animals were adapted to the technique and trained for three successive days earlier to the experimental procedure.

### 2.3. Blood Collection, Histology, Immunohistochemistry, and Biochemical Analysis

After the measurement of SBP, mice were anesthetized by intraperitoneal injection of sodium pentobarbital at a dose of 45 mg/kg, and then, the blood was collected from the inferior vena cava in citrate solution (3.2%). A sample was used for platelet count in a VET ABX Micros with mouse card (ABX, Montpellier, France), and the rest was spun at 4°C for 15 min at a speed of 900 *g*. The plasma samples acquired after centrifugation were kept at -80°C awaiting analysis.

The kidneys and hearts were excised following sacrifice, washed with ice-cold saline, blotted with filter paper, weighed, and fixed with 10% buffered formalin for 24 h. The latter was followed by dehydration in cumulative concentrations of C_2_H_5_OH, cleared with xylene, and embedded in paraffin. Sections of 3 *μ*m were prepared from paraffin blocks and stained with hematoxylin and eosin. These were examined by light microscopy by a histopathologist who contributed in this study (SA).

Concerning the detection of Nrf2 by immunohistochemistry, 5 *μ*m heart sections were made ready and mounted on aminopropyltriethoxysilane-coated slides. Subsequent to dewaxing with xylene and rehydrating with graded alcohol, slides were put in a 0.01 M citrate buffer solution (pH = 6.0) and the pretreatment processes to unmask the antigens were accomplished in a water bath at 95°C (30 min). Then, the sections were treated for 30 min with peroxidase block followed by protein block for 30 min. After that, sections were incubated for one hour at room temperature (RT) with anti-Nrf2 (Rabbit Polyclonal, 1 : 300, Abcam, USA). Following the conjugation with primary antibodies, sections were incubated with secondary antibody (EnVision^TM^ Detection System, DAKO, Agilent, USA) for 20 min at RT followed by DAB chromogen (EnVision^TM^ Detection System, DAKO, Agilent, USA) addition and counter staining achieved with hematoxylin. Suitable positive controls were utilized. Regarding the negative control, the primary antibody was not supplemented to sections. Both controls (negative and positive) were utilized in each set of slides which were stained (not shown in figures). The heart tissue immunohistochemical staining was scored according to the % of staining of heart muscles and endothelial cells of each section of the heart [18].

The concentrations of urea and creatinine in plasma and creatinine in urine were spectrophotometrically measured using commercial kits (Roche Diagnostics, Indianapolis, IN, USA). ELISA kits were utilized to quantify the levels of kidney injury molecule-1 (KIM-1) and neutrophil gelatinase-associated lipocalin (NGAL) in the urine (R&D Systems, MN, USA).

### 2.4. Assessment of Markers of Injury, Inflammation, and Oxidative Stress in Heart Homogenates

Preparation of heart homogenates for the assessment of markers of injury inflammation and oxidative stress was achieved as previously reported [19]. The levels of troponin I (Life Diagnostics, West Chester, PA, USA), tumor necrosis factor *α* (TNF-*α*; R&D Systems, Minneapolis, MN, USA), interleukin-1*β* (IL-1*β*; R&D systems, Minneapolis, MN, USA), 8-isoprostane (Cayman Chemicals, Michigan, USA), malondialdehyde (MDA; Sigma-Aldrich Fine Chemicals, St. Louis, MO, USA), superoxide dismutase (SOD; Cayman Chemicals, Michigan, USA), and catalase (Cayman Chemicals, Michigan, USA) were assayed according to the protocols described by the respective manufacturers.

### 2.5. DNA Damage Evaluation by COMET Assay

In a separate set of mice, the hearts and kidneys were removed from each mouse immediately after sacrifice and processed for the quantification of DNA injury by COMET assay according to a previously reported technique [17, 20].

### 2.6. *In Vivo* Experimental Pial Cerebral Microvessel Thrombosis Model

*In vivo* pial arteriolar and venular thrombogenesis was assessed on day 29 of the experiment in control and adenine-treated mice according to a previously described technique [17, 21].

### 2.7. *In Vitro* Assessment PT and aPTT

On day 29, all animals were anesthetized, and the blood was withdrawn from the inferior vena cava and placed in citrate solution (3.2%) (ratio of the blood to anticoagulant: 9 : 1). The PT was assayed on freshly collected platelet-poor plasma with human relipidated recombinant thromboplastin (Recombiplastin; Instrumentation Laboratory, Orangeburg, NY, USA) along with a coagulometer (MC 1 VET, Merlin, Lemgo, Germany) [22]. The aPTT was assessed with the automated aPTT reagent from bioMerieux (Durham, NC, USA) with the identical coagulometer [22].

### 2.8. Statistics

All statistical analyses were carried out using GraphPad Prism Software version 7. To assess whether the measured parameters were normally distributed, the Shapiro-Wilk normality test was first used. Normally distributed data were tested using the unpaired *t*-test for differences between the two groups. Data which were not normally distributed (TNF-*α*, IL-1*β*, MDA, and thrombotic occlusion time in venules) were tested using the Mann-Whitney test for differences between groups. All the data in figures and table were expressed as the mean ± SEM. *P* values < 0.05 are considered significant.

## 3. Results

### 3.1. Renal Endpoints

**Figure 1:**
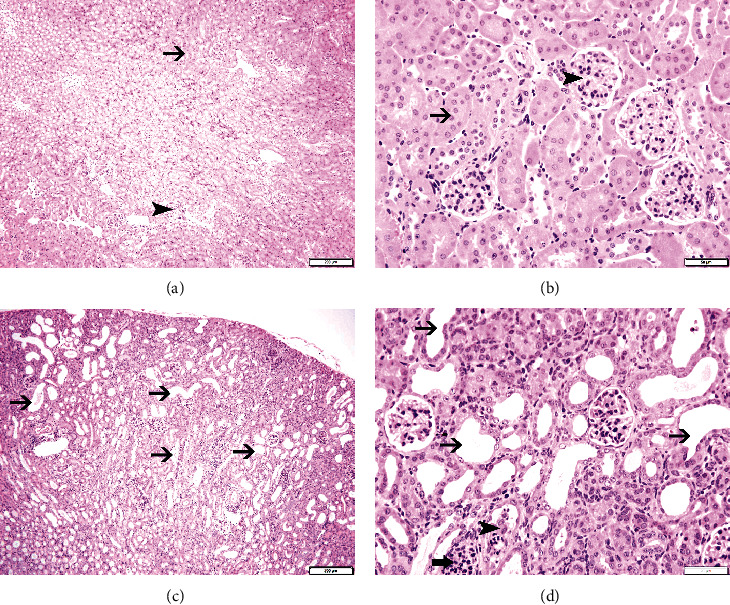
Representative light microscopy sections of kidney tissues of control mice and those given with adenine mixed in the feed (0.2% *w*/*w*, for four weeks), stained with H&E. (a, b) The control group shows unremarkable morphologic changes in renal tubules (thin arrow) and glomeruli (arrowhead). (c, d) The adenine-treated group shows dilatation of the renal tubules (thin arrow), tubular damage (arrowhead), and neutrophil polymorph infiltration of the affected tubules (thick arrow). Scale bars in (a, c): 200 *μ*m and (b, d): 50 *μ*m.

Tables 1 and 2 depict data related to physiological and biochemical parameters assessed in control and adenine-treated mice. Adenine treatment significantly decreased the body weight (*P* < 0.001) and significantly increased water intake (*P* < 0.001), urine volume (*P* < 0.01), and relative kidney weight (*P* < 0.05) when compared with control mice (Table 1).  Table 2 depicts a significant increase in plasma urea (*P* < 0.01) and creatinine (*P* < 0.05) concentrations, and a significant decrease in creatinine clearance (*P* < 0.001) in the adenine group. Moreover, adenine treatment induced a significant increase in the urinary concentrations of KIM-1 (*P* < 0.001) and NGAL (*P* < 0.0001) and DNA damage (*P* < 0.0001) assessed by COMET assay (Table 2). Likewise, the histological analysis of the kidneys collected from the adenine-treated group showed the presence of tubular dilation and damage and neutrophilic influx (Figure 1).

### 3.2. Cardiovascular Endpoints

**Figure 2:**
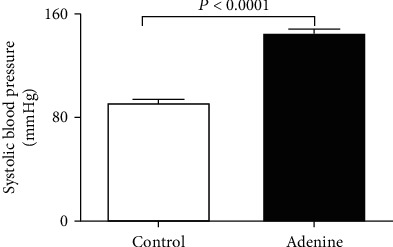
Systolic blood pressure in control mice and those given with adenine mixed in the feed (0.2% *w*/*w*, for four weeks). Mean ± SEM (*n* = 8 in each group).

Figure 2 shows that adenine treatment induced a significant increase in SBP (*P* < 0.0001).

**Figure 3:**
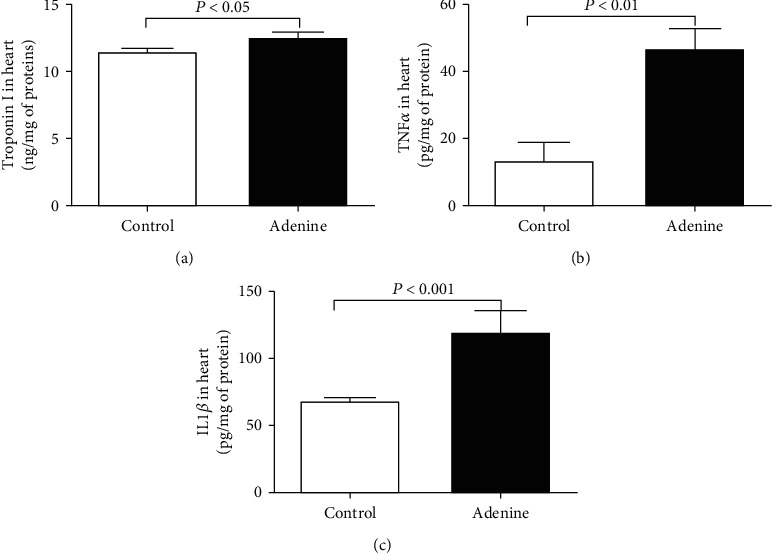
Troponin I (a), tumor necrosis factor-*α* (TNF-*α*) (b), and interleukin-1*β* (c) concentrations in heart homogenates of control mice and those given with adenine mixed in the feed (0.2% *w*/*w*, for four weeks). Mean ± SEM (*n* = 7‐8 in each group).

Figure 3 illustrates the effect of adenine administration on the concentrations of the marker of cardiac injury, troponin I, and the proinflammatory cytokines TNF-*α* and IL-1*β*. Compared with the control group, the concentrations of troponin I (*P* < 0.05), TNF-*α* (*P* < 0.01), and IL-1*β* (*P* < 0.001) were significantly increased in the heart homogenates of the adenine-treated group.

**Figure 4:**
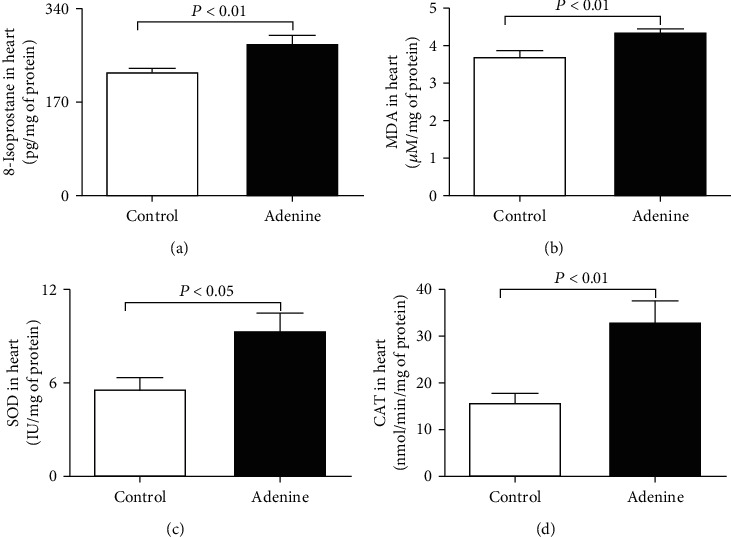
8-Isoprostane (a), malondialdehyde (MDA) (b), superoxide dismutase (SOD) (c), and catalase (CAT) (d) levels in heart homogenates of control mice and those given with adenine mixed in the feed (0.2% *w*/*w*, for four weeks). Mean ± SEM (*n* = 8 in each group).

Figure 4 shows the impact of adenine treatment on the levels of markers of lipid peroxidation including 8-isoprostane and MDA and the activities of the antioxidants SOD and catalase. Compared with the control group, the levels of 8-isoprostane (*P* < 0.01), MDA (*P* < 0.01), SOD (*P* < 0.05), and catalase (*P* < 0.01) were found to be markedly elevated in adenine-treated mice (Figure 4).

**Figure 5:**
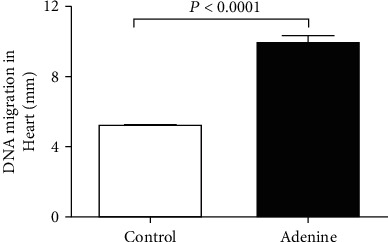
DNA migration (mm) in the heart tissues assessed by COMET assay in control mice and those given with adenine mixed in the feed (0.2% *w*/*w*, for four weeks) (a). Data are presented as the means ± SEM (*n* = 5).

The assessment of DNA damage by COMET assay in the heart of control and adenine-treated mice is shown in Figure 5. Compared with the control group, adenine administration induced a significant augmentation of heart DNA injury (*P* < 0.0001).

**Figure 6:**
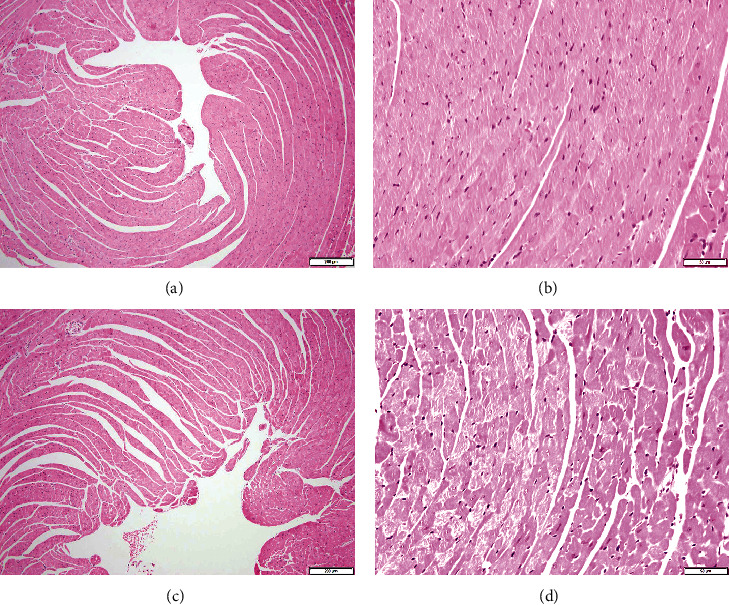
Representative light microscopy sections of heart tissues of control mice and those given with adenine mixed in the feed (0.2% *w*/*w*, for four weeks), stained with H&E. (a, b) The control group shows unremarkable heart morphology and architecture. (c, d) The adenine-treated group shows unremarkable heart morphology and architecture. Scale bars in (a, c): 200 *μ*m and (b, d): 50 *μ*m.

**Figure 7:**
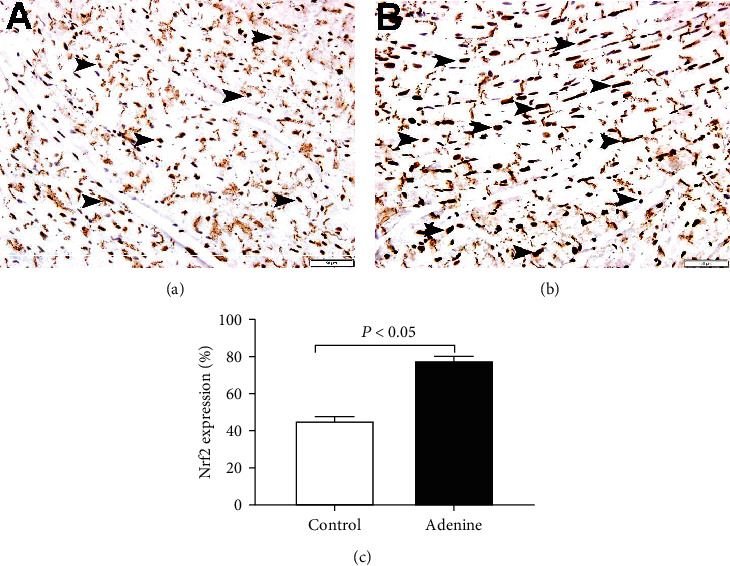
Immunohistochemical analysis of the heart tissue sections for the detection of nuclear factor erythroid-derived 2-like 2 (Nrf2) in control mice and those given with adenine mixed in the feed (0.2% *w*/*w*, for four weeks). (a) Representative section of the heart of control mice showing mild nuclear expression of Nrf2 by cardiomyocytes (arrow). (b) Representative section of the heart of adenine-treated mice showing a significant increase of nuclear expression of Nrf2 by cardiomyocytes (arrow). (c) Semiquantitative assessment of the % immunohistochemical staining of the heart tissue for Nrf2 in control mice and those given with adenine mixed in the feed. Data are presented as the means ± SEM (*n* = 6). Scale bars in (a, b): 50 *μ*m.

Light microscopy analysis of the heart sections stained with H&E obtained from control mice exhibited normal structure (Figure 6). Following adenine treatment, no morphological changes have been observed in the hearts collected from the adenine group (Figure 6). However, the immunohistochemistry analysis of the heart revealed the presence nuclear expression of Nrf2 by cardiomyocytes in the heart sections of all groups (Figure 7), with different intensity and distribution. The control group shows mild nuclear expression of Nrf2 by cardiomyocytes (Figure 7). The adenine-treated group showed a significant increase (*P* < 0.05) in the expression of Nrf2 by cardiomyocytes when compared to the control group (Figure 7).

**Figure 8:**
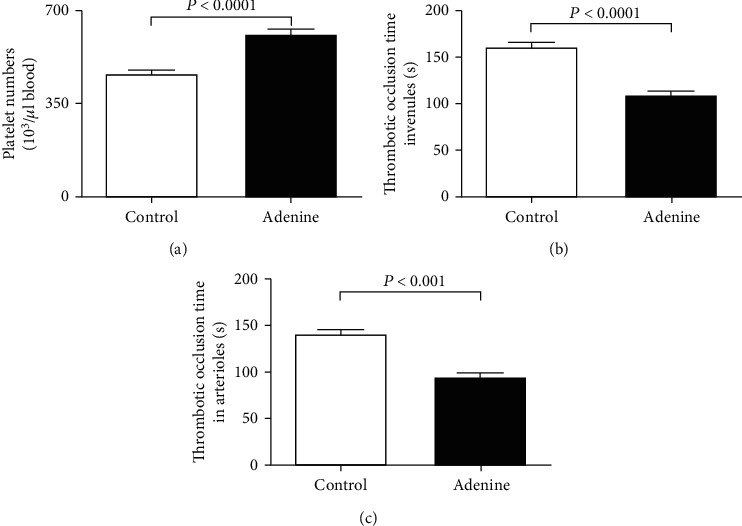
Circulating platelet numbers (a) and thrombotic occlusion time in pial arterioles (b) and venules (c) in control mice and those given with adenine mixed in the feed (0.2% *w*/*w*, for four weeks). Mean ± SEM (*n* = 7‐8 in each group).

Figure 8 shows the effects of adenine on the number of circulating platelets and the thrombotic events in pial microvessels *in vivo*. Compared with the control group, the number of circulating platelets was significantly augmented (*P* < 0.0001), and thrombotic occlusion time measured in pial arterioles significantly shortened (*P* < 0.0001) in adenine-treated mice (Figure 8(a) and Figure 8(b), respectively). Similarly, in pial venules of adenine-treated mice, the thrombotic occlusion time was also significantly shortened (*P* < 0.001) compared with the control group, indicating a prothrombotic effect of adenine treatment (Figure 8(c)).

**Figure 9:**
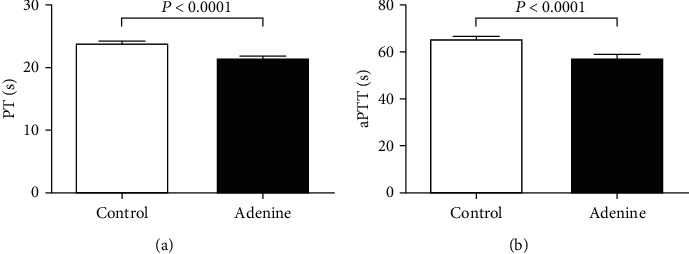
Prothrombin time (PT) (a) and activated partial thromboplastin time (b) in plasma of control mice and those given with adenine mixed in the feed (0.2% *w*/*w*, for four weeks). Mean ± SEM (*n* = 7‐8 in each group).

Figure 9 depicts the impact of adenine administration on PT and aPTT. Compared with the control group, adenine treatment significantly shortened the PT (*P* < 0.0001; Figure 9(a)) and aPTT (*P* < 0.0001; Figure 9(b)).

## 4. Discussion

In the present study, we showed that feeding adenine to mice (0.2% *w*/*w* for four weeks) induced CKD which was associated with cardiac inflammation, oxidative stress, Nrf2 expression, and DNA damage. It also induced prothrombotic events *in vivo*.

Renal and cardiovascular functions are tightly related under physiological and pathophysiological situations [23, 24]. It is well-known that cardiovascular diseases are the main cause of death in patients with impaired renal function [23, 24]. Moreover, patients with CKD develop cardiovascular disease including hypertension, cardiac hypertrophy, and thrombotic complications [23, 24].

Experimental animal models using rats or mice have been widely utilized to enhance our knowledge about the pathophysiology of CKD and develop pharmacological interventions aiming at mitigating or averting renal damage [4]. Although the majority of CKD animals models do not simulate totally the intricacy of human CKD and its accompanying complications, the experimental CKD induced by adenine supplementation to feed of rats or mice have been shown to reproduce gastrointestinal, pulmonary, and cardiovascular complications seen in clinical situations [4, 7, 8, 12, 15, 25]. With respect to cardiovascular effects, it has been shown that adenine treatment in rats (0.75% *w*/*w* for four weeks) increased blood pressure and the absolute volume of left ventricle and it decreased volume density and absolute volume of myocardial capillaries [7, 25]. Moreover, Diwan et al. [4, 8] showed that adenine added in diet at 0.25% for 16 weeks mimicked the cardiovascular changes seen in humans with CKD, including elevation of blood pressure (which was significant at four weeks) along with hypertrophy of the left ventricle and augmentation in interstitial and perivascular inflammation and fibrosis resulting in augmented stiffness of the left ventricular. However, the mechanisms underlying the cardiovascular events seen in CKD are not fully understood.

In line with earlier reports describing the renal effects of adenine treatment in mice [6, 12, 14], we found here that the body weight and creatinine clearance were reduced, whereas the water intake, urine volume, relative kidney weight, the plasma concentrations of urea and creatinine, the urinary concentrations of KIM-1 and NGAL, and renal DNA damage were significantly elevated compared with the control group. Moreover, histological analysis of the kidneys collected from the adenine-treated group showed the presence of tubular dilation and damage and neutrophilic influx.

It has been previously shown that adenine-treated rats (0.25% or 0.75% *w*/*w*) induce an increase of blood pressure at four-week time point and continues up to 16 weeks [8, 25, 26]. Likewise, adenine administration in mice (0.2% for four weeks) induced a significant increase of blood pressure [6, 14]. The data of the present study confirmed the elevation of SBP of mice given with adenine and further showed a significant increase of troponin I, a biomarker of myocardial damage, and two proinflammatory cytokines including TNF-*α* and IL-1*β* in heart homogenates. The latter findings indicate the occurrence of myocardial injury and cardiac inflammation. Both inflammation and oxidative stress are concurrently found to be elevated in cardiovascular diseases, and each one can be readily triggered and potentiated by the other one [27, 28]. Recently, inflammation and oxidative stress have attracted much interest as crucial pathophysiological players in the cause and progression of various cardiovascular diseases [27, 28]. Despite the fact that reactive oxygen species exert signalling functions under physiological condition, a disproportionate and decontrolled generation of these molecules may trigger oxidative stress and cardiomyocyte injury [27, 28]. Therefore, to gain more insights into the mechanisms underlying the observed cardiac injury and inflammation, we measured various markers of oxidative stress in heart homogenates, including 8-isoprostane, MDA, SOD, and catalase. Our data showed that the hearts of adenine-treated mice showed a significant increase of markers of lipid peroxidation, namely, 8-isoprostane and MDA, and the antioxidant enzymes SOD and catalase. The latter indicate the occurrence of oxidative stress in the heart, and the increase of antioxidants suggests an ongoing compensatory mechanisms taking place in the heart and aiming at mitigating the injurious effects of oxygen radicals. Furthermore, besides heart inflammation and oxidative stress, we, presently, found a significant increase in cardiac DNA damage. These results indicate that the treatment with adenine caused oxidative stress *milieu* which in turns induced DNA injury. As far as we are aware, these findings have not been reported before. Using the same model of adenine-induced CKD in mice, we have recently reported the occurrence of lung oxidative stress and DNA damage [12].

Despite the occurrence of inflammation, oxidative stress, and DNA damage seen in the adenine group, the histological analysis of the hearts revealed a lack of clear morphologic alterations in H&E-stained sections. This effect can be related to the duration of adenine treatment (4 weeks at 0.2% *w*/*w*) applied in this study. Moreover, the apparent absence of morphological alteration is not in disagreement with the occurrence of biochemical changes shown presently in heart tissue of mice treated with adenine (Figures 3–5). The latter biochemical alterations can plausibly pave the way of morphological change of the heart which may be seen after treatment with adenine for a longer period of time [29]. In fact, in rats exposed to adenine for sixteen weeks at 0.25 *w*/*w*, various cardiac morphological changes have been reported encompassing compromised vascular responses, elevated left ventricular stiffness, and augmented left ventricular mass [8]. The immunohistochemistry analysis of the heart obtained from the adenine-treated group revealed a significant increase in the nuclear expression of Nrf2 by cardiomyocytes. Nrf2 is an important transcription factor involved in antioxidant enzyme activation after the manifestation of oxidative stress [30, 31]. Following the occurrence of oxidative stress, Nrf2 is freed from the regulatory Keap1-Nrf2 complex and moves from the cytoplasm to the cell nucleus, where it attaches to the antioxidant response element, a regulatory enhancer region within gene promoters [32]. This attachment triggers the production of antioxidant enzymes that play a protective role against oxidative stress-induced cell injury [32]. We have recently demonstrated the increase of expression of Nrf2 in the lung of mice treated with adenine [12]. Moreover, Nrf2 expression was found to increase in the heart of mice exposed to waterpipe tobacco smoke, and that the treatment with the antioxidant gum Arabic potentiated Nrf 2 expression [18].

Patients with CKD present various defects in hemostasis and coagulation including the elevation platelet aggregation and von Willebrand factor activity, and the plasma concentrations of D-dimer, fibrinogen, and plasminogen activator inhibitor that prevents the activation of the fibrinolytic system [33, 34]. The latter effects induce increase of thrombogenicity in CKD [33, 34]. On the other hand, as the CKD progresses to a greater extent, the bleeding risk augments in relation to platelet defect [33, 34]. Experimental studies using rat and mouse model of CKD induced by 5/6 ablation/infarction showed an increase of platelet activity and a prothrombotic tendency assessed 4 weeks after the surgery [10, 11]. Conversely, a recent study has shown a prolongation of tail bleeding time and delay in thrombus formation in cremaster arterioles following vascular injury in mice with CKD induced by 5/6^th^ nephrectomy or fed with 0.25% adenine (for two weeks) [9]. In the present work, similar to our previous studies using the same dose and duration of treatment (0.2% for four weeks), we did not notice any mortality related to the adenine treatment [6, 12, 14]. Our data show a significant increase in the number of circulating platelet. In patients with CKD, relative thrombocytosis has been associated with the severity of cardiovascular disease [35, 36]. Interestingly, our data also showed a significant shortening in the thrombotic occlusion time in pial arterioles and venules *in vivo* indicating an increased thrombogenicity. Additionally, we assessed the PT and aPTT in plasma of control and adenine-treated mice. PT evaluates the production of the fibrin clot through the activity of the extrinsic and common coagulation pathways, and aPTT assesses the activity of the intrinsic and common pathways of coagulation. We found a significant shortening of the PT and aPTT in the plasma of the adenine-treated group, demonstrating a propensity to hypercoagulability and confirming our in vivo findings. The discrepancy between our study and that of Makhloufi et al. [9] could be ascribed to the difference in the dose and duration of treatment of adenine (4 weeks at 0.2% in our study versus 2 weeks at 0.25%). In the latter study, the reason why the duration of treatment with adenine was limited to 2 weeks was related to high animal mortality observed at 4 weeks of treatment [9]. Additional studies are needed to understand the reason of this discrepancy and to further investigate the mechanism of increased thrombotic tendency in adenine-treated mice.

In conclusion, our data show that administration of adenine in mice induced CKD which is associated with cardiac inflammation, oxidative stress, Nrf2 expression, and DNA damage. It also induced prothrombotic events *in vivo*. Further studies are required to establish whether adenine can have direct harmful effects on the heart.

## Figures and Tables

**Table 1:** Daily water intake, urine volume, body weight change, and relative kidney weight in control and adenine-treated mice.

Parameters/group	Control	Adenine
Water intake (ml)	9.08 ± 0.55	22.83 ± 2.45^∗∗∗^
Urine volume (ml)	3.11 ± 0.05	14.32 ± 0.926^∗∗^
Body weight (% change)	6.25 ± 0.97	−22.26 ± 0.80^∗∗∗^
Relative kidney weight (g)	1.14 ± 0.02	1.23 ± 0.03^∗^

Values in the table are presented as the mean ± SEM (*n* = 6 − 8). Adenine was added to the feed at a concentration of 0.2% *w*/*w*, for 4 weeks. ^∗^*P* < 0.05, ^∗∗^*P* < 0.01, and ^∗∗∗^*P* < 0.001 (control vs. adenine group).

**Table 2:** Plasma concentration of urea and creatinine and creatinine clearance, and urinary concentration of kidney injury molecule-1 (KIM-1) and neutrophil gelatinase-associated lipocalin (NGAL), and kidney DNA damage in control and adenine-treated mice.

Parameters/group	Control	Adenine
Urea (mmol/l)	4.05 ± 0.15	8 ± 1.08^∗∗^
Creatinine (*μ*mol/l)	9.1 ± 0.72	17.63 ± 3.72^∗^
Creatinine clearance (ml/min)	0.50 ± 0.05	0.13 ± 0.03^∗∗∗^
KIM-1 (pg/ml)	86.25 ± 4.32	762.3 ± 54.58^∗∗∗^
NGAL (pg/ml)	4,477 ± 262	6,897 ± 308.4^∗∗∗∗^
DNA migration in kidney (mm)	5.23 ± 0.10	10.43 ± 0.05^∗∗∗∗^

Values in the table are presented as the mean ± SEM (*n* = 5 − 8). Adenine was added to the feed at a concentration of 0.2% *w*/*w*, for 4 weeks. ^∗^*P* < 0.05, ^∗∗^*P* < 0.01, ^∗∗∗^*P* < 0.001, and ^∗∗∗∗^*P* < 0.0001 (control vs. adenine group).

## Data Availability

The data that support the findings of this study are available from the corresponding author, Abderrahim Nemmar, upon reasonable request.
